# We are all in this together: Understanding organ crosstalk in the pathogenesis of acute respiratory distress syndrome

**DOI:** 10.1002/pul2.12418

**Published:** 2024-07-19

**Authors:** Dustin R. Fraidenburg

**Affiliations:** ^1^ Department of Medicine, Division of Pulmonary, Critical Care, Sleep and Allergy University of Illinois Chicago Chicago Illinois USA

I am writing this as I return from a conference of over 10,000 professionals dedicated primarily to the research and clinical care of respiratory disease. It is wonderful to reconnect with friends, colleagues, and mentors; to network and form new professional relationships with like‐minded researchers and clinicians; and to see all the wonderful work and scientific breakthroughs that are occurring in our field; but I cannot help but feel like this design is isolating and restrictive to our more lofty goals. In the manuscript by DeWolf and colleagues entitled, “Human pulmonary microvascular endothelial cells respond to damage‐associated molecular patterns (DAMPs) from injured renal tubular cells” the authors identify important organ crosstalk with a molecular signal released from renal tubular cells that elicits endothelial activation and dysfunction in the lung.^1^ After necrotic injury to renal tubular epithelial cells, the authors identify numerous proteins released into the supernatant which are classified as DAMPs through their known interaction with NOD like receptors and toll like receptors. They show that these DAMPs activate important signaling pathways in lung microvascular endothelial cells, are associated with endothelial dysfunction, and cause increased endothelial barrier permeability. The authors conclude that lung microvascular endothelial cells respond to DAMPs derived from renal tubular epithelial cells during injury through pattern recognition receptors as a mechanism for crosstalk between the lung and kidney.

As intensivists, our practice is not limited to one organ system and we often encounter multiorgan dysfunction; requiring careful consideration of the role of each organ independently and in concert within the body to provide the best supportive and therapeutic measures. Acute respiratory distress syndrome (ARDS) is characterized by lung disease and respiratory failure, yet we know that direct injury to the lung through pneumonia, aspiration, inhalation, or lung trauma is not the whole story.^2^ Important causes of ARDS manifest outside of the pulmonary system with risk factors such as non‐pulmonary sepsis, pancreatitis, transfusion‐related complications. While understanding these risk factors has allowed for increased attention and surveillance for ARDS in these cases, the precise mechanisms are mostly elusive. When we think about distant organ dysfunction as the etiology of ARDS, acute pancreatitis becomes an obvious example. Understanding the relationship of acute pancreatitis to the development of ARDS has resulted in various risk profiles and calculators.^3^, ^4^ It has long been known that markers of endothelial dysfunction with elevated von Willebrand factor and a dysregulated immune system with reduction in shed/soluble L‐selectin, are associated with severe pancreatitis with high risk for ARDS.^5^, ^6^ Despite this understanding and clear association, there is little mechanistic work that has been done to understand the particular factors at play in the development, progression, and the potential recovery phase of ARDS in acute pancreatitis. I cannot help but think that this is because the pancreatitis experts and ARDS experts have turned to their separate professional silos.

Both the lungs and kidneys have been important focus of research in critical care separately, but it has not been lost on researchers that there is important interactions that impact disease pathogenesis, clinical deterioration, and mortality in this vulnerable.^7^, ^8^, ^9^ The Acute Disease Quality Initiative working group identified broad categories of shared mechanisms between acute kidney injury and respiratory failure including inflammation, fluid shifts, infection, immune dysfunction, heart dysfunction, hypoxemia, as well as intrathoracic and intra‐abdominal pressure changes during mechanical ventilation.^10^ A focal point of this consensus statement is the significant knowledge gaps identified in these interactions and crosstalk along with a call for numerous areas of further research and investigation. This publication in *Pulmonary Circulation* journal is most definitely a step in the right direction. Supernatant from the necrotic renal tubular epithelial cells, which is rich in DAMPs, caused significant injury to the lung microvasculature, recapitulating the injury leading to ARDS. This broad approach certainly demonstrates the importance of organ crosstalk and injury, yet I cannot help but wonder if there are particular DAMP proteins floating in that supernatant which hold a key to changing the paradigm of ARDS therapy. Highlighting the importance of narrowing in on specific DAMP entities, extracellular nicotinamide phosphoribosyltransferase is shown to be an important DAMP that serves as a biomarker for early diagnosis of ARDS associated with acute pancreatitis as well as a druggable target under investigation in an ongoing clinical trial (NCT05938036).^11^ As our understanding and precision grow, I imagine that we will better distinguish these specific patterns of injury, such that we can identify early markers of ARDS risk and also develop targeted therapeutics. I applaud the authors here for moving the bar; I hope that their continued work, as well as the work of those that see this publication, will forge new collaborations, will step outside of the professional silo and potentially impact the care for not just one organ but many, because we are all in this together.

## AUTHOR CONTRIBUTIONS

Dustin R. Fraidenburg conceived and contributed to writing the manuscript.

## CONFLICT OF INTEREST STATEMENT

The author declares no conflict of interest.

## ETHICS STATEMENT

This work does not involve human subjects, animal experimentation, or cell lines.
